# Should all patients with hypertension be worried about developing severe coronavirus disease 2019 (COVID-19)?

**DOI:** 10.1186/s40885-021-00161-7

**Published:** 2021-01-15

**Authors:** Ramin Hosseinzadeh, Mohammad Ali Sheikh Beig Goharrizi, Mansour Bahardoust, Akbar Ghorbani Alvanegh, Mohammad Reza Ataee, Mehdi Bagheri, Ensiyeh Shabani Navidiyan, Seyed Reza Hosseini Zijoud, Mohammad Heiat

**Affiliations:** 1 Baqiyatallah Research Center for Gastroenterology and Liver Diseases, Baqiyatallah University of Medical Sciences, Tehran, Iran; 2 Atherosclerosis Research Center, Baqiyatallah University of Medical Sciences, Tehran, Iran; 3 Department of Epidemiology, School of Public Health, Shahid Beheshti University of Medical Sciences, Tehran, Iran; 4 Human Genetics Research Center, Baqiyatallah University of Medical Sciences, Tehran, Iran; 5 Applied Microbiology Research Center, Systems Biology and Poisonings Institute, Baqiyatallah University of Medical Sciences, Tehran, Iran; 6 Chemical Injuries Research Center, Systems Biology and Poisonings Institute, Baqiyatallah University of Medical Sciences, Tehran, Iran; 7 Student Research Committee, Baqiyatallah University of Medical Sciences, Tehran, Iran; 8 Clinical Research Development Unit, Imam Hossein Hospital, Shahid Beheshti University of Medical Sciences, Tehran, Iran

**Keywords:** Hypertension, Risk factor, Severe COVID-19

## Abstract

**Background:**

Hypertension, the most common comorbidity among coronavirus disease 2019 (COVID-19) patients, is accompanied by worse clinical outcomes, but there is lack of evidence about prognostic factors among COVID-19 patients with hypertension. We have come up with some prognostic factors to predict the severity of COVID-19 among hypertensive patients. In addition, epidemiologic, clinical and laboratory differences among COVID-19 patients with and without underlying hypertension were evaluated.

**Methods:**

Medical profiles of 598 COVID-19 cases were analyzed. Patients were divided into two comparative groups according to their positive or negative history of hypertension. Then, epidemiologic, clinical, laboratory and radiological features and also clinical outcomes were compared.

**Results:**

176 (29.4%) patients had underlying hypertension. Diabetes was significantly higher in hypertensive group [72 (40.9%) vs 76 (18%)] (*P*-value: 0.001). Cardiovascular and renal disorders were significantly higher in hypertensive patients. (*P*-value: 0.001 and 0.013 respectively). In COVID-19 patients with hypertension, severe/critical types were significantly higher. [42(23.8%) vs. 41(9.7%)], (*P*-value: 0.012). In the logistic regression model, Body mass index > 25 (OR_Adj_: 1.8, 95% CI: 1.2 to 2.42; *P*-value: 0.027), age over 60 (OR_Adj_: 1.26, 95% CI: 1.08 to 1.42; *P*-value: 0.021), increased hospitalization period (OR_Adj_: 2.1, 95% CI: 1.24 to 2.97; *P*-value: 0.013), type 2 diabetes (OR_Adj_: 2.22, 95% CI: 1.15 to 3.31; P-value: 0.001) and chronic kidney disease (OR_Adj_: 1.83, 95% CI: 1.19 to 2.21; P-value: 0.013) were related with progression of COVID-19.

**Conclusion:**

Hypertensive patients with Age > 60-year-old, BMI > 25 Kg/m^2^, CVD, diabetes and chronic kidney disease are associated with poor outcomes in those with COVID-19 infection.

## Introduction

The COVID-19 pandemic has involved millions of people and had thousands of victims globally. To the moment of writing this paper (May 5th, 2020), 4,894,278 cases and 320,189 deaths were reported [1]. COVID-19 infection is caused by severe acute respiratory syndrome corona virus-2 (SARS-CoV-2), a member of coronaviridae family, believed to able to spread to human from reservoirs like bats [2].

Due to their large genome, members of coronaviridae family have a great potential to initiate new epidemics as a result of gene mutation. This family of viruses is morphologically known by a crown like protein spike on their envelope [3].

While common symptoms of COVID-19 infection which are cough, fever, fatigue and myalgia can be seen after about 5 days of incubation period, uncommon symptoms such as dyspnea, chest pain, chest tightness, diarrhea and vomiting can also be seen during the time course of infection [3–12].

The main causes of death in COVID-19 infection are acute respiratory distress syndrome (ARDS), renal and cardiac failure [13]. Global mortality rate of COVID-19 infection is estimated about 6%(5.7%) [7].

It is acknowledged that people with comorbidities are at a greater risk of getting infected with SARS-CoV-2 and developing severe disease. Hypertension is the most common comorbidity among COVID-19 patients which is accompanied by higher risk of infection and worse outcomes and prognosis [13–18]. The fact that hypertension is the most common comorbidity in COVID-19 patients is not a great surprise because the frequency and severity of the infection is higher in elderly and hypertension is seen frequently in old individuals and as a result, hypertension can be seen in a lot of COVID-19 cases. However it remains unclear whether uncontrolled blood pressure is a risk factor for getting infected with COVID-19 and developing severe disease or not [18].

A meta-analysis demonstrated that patients with underlying cardiovascular disease like hypertension are more susceptible to MERS-CoV infection [19] and some studies have shown that patients with cardiovascular disease especially hypertension are at a greater risk for getting infected with COVID-19 and developing more severe disease but further investigations are needed in this area [20].

Although hypertension is the most common comorbidity among COVID-19 patients and some studies have shown its association with worse outcomes [13–16, 18], there in not enough data available about prognostic factors among COVID-19 patients with underlying hypertension to predict possible outcomes among them, So in the present study we aimed to evaluate epidemiologic, clinical and laboratory differences between COVID-19 patients with and without underlying hypertension and came up with some prognostic factors to predict the severity of the disease and other possible outcomes among patients with pre-existing hypertension.

## Method

### Study design and setting

This retrospective study was approved by the review board of our institute IR.BMSU.REC.1399. 244 and informed consent was obtained from the patients to use their medical information. In this retrospective analytical study, medical profiles of 598 cases with COVID-19 pneumonia who were admitted to Baqiyatallah Hospital in Tehran – Iran were evaluated. Data collection was done via checklists filled with information extracted from patients’ medical profiles. All patients had agreed to share their information to help studying the coronavirus outbreak on admission and filled consent forms. Patients’ information including demographic data (age, gender, history of smoking, history of alcohol consumption, history of travel in past 2 weeks, history of contact with COVID-19 patents in past 2 weeks, history of hospital visit in past 2 weeks and history of having underlying disease), clinical and laboratory data (fever, gastrointestinal symptoms, biochemical tests, liver function tests, respiratory tests, blood work, radiological findings and CT-scan) was collected. Information about clinical outcomes (increase in hospitalization period, mortality and need to readmission) was also collected. According to previous data, the median of hospitalization period among COVID-19 patients was 14 days in china and 5 days outside china [21] so, a period more than 14 days was defined as increased hospital stay. Patients with incomplete medical profiles, patients who were not receiving any kind of anti-hypertensive medications and patients who were receiving any corticosteroids were excluded. Then, patients were divided into two comparative groups according to their positive or negative history of hypertension. According to the guideline for the diagnosis and management of hypertension in adults—2016 [22],Systolic blood pressure > 140mmhg and diastolic BP > 90 mmhg were set as hypertension cutoff. Final hypertension diagnosis was made by an internal medicine specialist. All hypertensive patients of this study were receiving anti-hypertensive medications. Definite diagnosis of COVID-19 and differentiating it from other viruses like H1N1, H3N2 and H7N9 influenza, respiratory syncytial virus, parainfluenza virus, adenovirus, SARS-CoV and MERS-CoV was done by RT-PCR test and CT-scan results. Finally, epidemiologic, clinical, laboratory and radiological features and also clinical outcomes were compared between patients with and without hypertension. All COVID-19 patients can be divided into one of the clinical stages described below; mild: minimal clinical symptoms and no pneumonia on imaging; moderate: showing clinical symptoms like fever and respiratory manifestations accompanying pneumonia on imaging; severe; cases with at least one criterion of the following criteria:(1) respiratory rate ≥ 30 times/minute with shortness of breath; (2) arterial partial pressure of oxygen/fraction of inspiration of oxygen≤300 mmHg;(3) finger oxygen saturation ≤ 93% in the resting state; and (4) lesion progression of more than 50% in 24–48 h on the chest imaging. Critical: patients with at least one on the following conditions: (1) respiratory failure requiring mechanical ventilation; (2) shock; and (3) failure of other organs, and the need for intensive care unit admission [23, 24].

### Statistical analysis

The collected data was analyzed by SPSS version 22. Descriptive analysis and central tendency (mean and standard deviation) were used for baseline data. In order to compare quantitative variables in two groups, T test was used for data with normal distribution and Mann–Whitney U test was used for data without normal distribution. Chi-square test was performed to compare qualitative variables. Logistic regression Multivariate analysis was performed to determine most important risk factors of COVID-19 infection severity in hypertensive patients. Variables with *p* < 0.10 in the univariate analysis test were entered into a logistic regression multivariate analysis with the backward selection method. This part’s findings were reported as odds ratio (OR) with 95% confidence interval. *P*-value of < 0.05 was set as statistical significance cutoff.

## Results

In this study, 598 medical profiles of COVID-19 patients were analyzed. 176(29.4%) patients were hypertensive and 422(70.6%) were normotensive. Mean age was 58.21 ± 22.5 in hypertensive group and 56.21 ± 20.2 in normotensive group (*P*-value: 0.18). 115(65.3%) patients in hypertensive group and 289(68.5%) patients in normotensive group were men (*P*-value:0.78). Diabetes was seen more in hypertensive group.72(40.9%)hypertensive patients and 76(18%) normotensive ones were diabetic which was statistically significant(*P*-value:0.001). Cardiovascular disease was significantly higher in hypertensive group[63,(35.7%)] in comparison to normotensive group[44(10.3%)] (*P*-value:0.001). Renal disorders were significantly higher in hypertensive group [25(14.3%)]vs.[33(7.8%)] respectively (P-value:0.013). Severe/critical types were significantly higher in hypertensive group comparing to normotensive one, [42(23.8%) vs 41(9.7%)], (*P*-value:0.012). No significant difference was seen in other variables (history of exposure to COVID-19 patients in past 2 weeks, smoking, brain stroke, respiratory disease, cancer(P-value > 0.05), (Table 1).

**Table 1:** Comparison of demographic characteristics of patients with COVID-19 in two groups (hypertensive and normotensive patients***)***

Variable	hypertensive (N: 176)	Non-hypertensive (N:422)	***P***-value
Age (Year)	58.21 ± 22.5	56.15 ± 20.2	0.18
BMI (kg/m^2^)	28.62 ± 4.6	28.01 ± 4.3	0.13
Sex (male)	115(65.3%)	289(68.5%)	0.78
Education			0.65
• < diploma	57 (32.4%)	111(26.3%)	
• >Diploma	119(67.6%)	311 (73.7%)	
Exposure history	30(17%)	191 (45.2%)	0.45
• Contact with patients	118(67%)	250 (59.2%)	0.061
• Go hospital	30(17%)	87(20.6%)	0.23
• Public transportation	24(22.5%)	118(24.1%)	0.64
Comorbidities (positive)	108(61.3%)	249(59%)	0.75
Current smoker	4(2.2%)	12(2.8%)	0.92
Diabetes	72(40.9%)	76(18%)	0.001
CVD	63(35.7%)	44(10.3%)	0.001
MI	4(2.3%)	2(0. 5%)	0.061
Lung diseases	36(20.4%)	52(12.3%)	0.07
Asthma and Allergy	31(17.6%)	67 (15.9%)	0.45
Kidney diseases	25(14.3%)	33(7.8%)	0.013
Liver diseases	10(5.7%)	22(5.2%)	0.47
Cancer	4(2.3%)	8(1.9%)	0.68
Blood Group			
• A	55(31.3%)	139(32.8%)	0.21
• B	45(25.5%)	110(26.3%)	0.71
• AB	21(11.9%)	60(14.2%)	0.52
• O	55(31.3%)	113(26.7%)	0.31
Clinical type of COVID- 19 infection on admission			
Severe/Critical type (%)	42(23.8%)	41(9.7%)	0.012

MI: myocardial infarction, CVD: cardiovascular disease

### Clinical and laboratory features and clinical outcome

Univariate analysis showed that the number of patients with high fever (> 38.5) were significantly higher in hypertensive group [75(44.3%)] in comparison to normotensive one [125 (9.6%)], (P: 0.042). Similar results were observed about Dyspnea; [22(12.5%) vs. 20(4.5%)] (P: 0.023).

CT-scan findings showed that unilateral involvement was significantly lower in hypertensive group [25(14.2%)] in comparison with normotensive group [86(20.3%)], (*P* value 0.046). However no significant difference was seen in bilateral pulmonary involvement, multiple mottling and ground glass opacity between study groups (*P* > 0.05). Other clinical symptoms and laboratory results didn’t show significant difference between two groups (Table 2).

**Table 2:** Comparison of clinical symptoms, laboratory findings and clinical outcomes of patients with COVID-19 in two groups (hypertensive and normotensive patients***)***

Symptoms	Normal range	hypertensive (N: 176)	Non-hypertensive (N:422)	P value
Fever	N (%)			0.042
• ≥38.5 c		78(44.3%)	125(29.6%)	
• < 38.5		98(45.7%)	300(70.4%)	
Cough	N (%)	103 (58.5%)	257(60.9%)	0.38
Dysphonia	N (%)	105(59.6%)	261(61.8%)	0.37
Chest pain	N (%)	38(21.6%)	107(25.3%)	0.19
Dizziness	N (%)	19(10.7%)	56(13.2%)	0.18
Headache	N (%)	56(31.8%)	153(36.2%)	0.18
Weakness	N (%)	90(51.4%)	232(55.1%)	0.24
Rhinorrhea	N (%)	20(11.3%)	41(9.7%)	0.3
Myalgia	N (%)	62(35.2%)	154(36.5%)	0.61
Test Sense	N (%)	21(11.9%)	51(12%)	0.91
Shortness of breath	N (%)	22(12.5%)	20(4.7%)	0.023
Diarrhea	N (%)	34(19.3%)	84(19.9%)	0.76
Vomiting	N (%)	53(30.2%)	136(32.3%)	0.23
**Laboratory tests**				
Leucocytes (× 109/L)	4–10	7.61 ± 7.45	6.26 ± 3.08	0.14
Neutrophils (×109/L)	2–7	6.28 ± 0.65	5.23 ± .065	0.12
Lymphocytes (×109/L)	0.8–4	2.09 ± 1.68	2.06 ± 1.68	0.31
Platelets (×109/L)	125–450	164.2 ± 63.1	159.6 ± 75.2	0.12
Hemoglobin (g/L)	115–150	15.67 ± 2.7	14.8 ± 2.8	0.068
Hematocrit (%;)	33.35–50.8	44.15 ± 3.7	42.1 ± 4.3	0.23
Albumin (g/L)	40–55	44.15 ± 7.8	41.5 ± 6.5	0.063
Alt(U/L)	9–50	40.5 ± 24.6	39.6 ± 29.2	0.081
Ast(U/L)	15–40	36.1 ± 26.2	32.2 ± 25.1	0.038
Total bilirubin)	0–26	9.1 ± 3.1	8.6 ± 4.2	0.25
Serum sodium (mmol/L)	137–147	135.6 ± 15.5	134.1 ± 18.1	0.71
Serum potassium (mmol/L)	3.5–5.3	3.41 ± 2.4	3.31 ± 2.9	0.77
Glucose (mmol/L)	70–140	5.99 ± 2.15	5.01 ± 1.96	0.62
INR	0.85–1.15	1.41 ± 0.17	1.15 ± 0.13	0.28
CRP (mg/L) d	0–10	41.2 ± 32.1	39.8 ± 31.2	0.11
Chest X-ray/CT findings	N (%)			
• Normal		19(10.7%)	50(11.8%)	0.24
• Unilateral pneumonia		25(14.2%)	86(20.3%)	0.046
• Bilateral pneumonia		68 (38.6%)	156(36.9%)	0.12
• Multiple mottling and ground-glass opacity		64(36.5%)	159(31%)	0.071
**Clinical outcome**				
Increased hospital stays	N (%)	41(22.9%)	46(10.9%)	0.015
Death	N (%)	16(9.1%)	25(5.9%)	0.11
Readmission	N (%)	20(11.3%)	42(9.9%)	0.28

PCR: Polymerase chain reaction, ALT: Alanine aminotransferase AST: Aspartate transaminase, BS: blood sugar, INR: International normalized ratio, CRP: c-reactive protein

Comparison of clinical outcomes of COVID-19 between our study groups showed that 41(22.9%) of hypertensive and 46(10.9%) of normotensive patients needed longer hospital stay which was statistically significant (*P*-value: 0.015). Mortality and need to readmission showed no significant difference between two groups (*P* value > 0.05), (Table 2). Cause of death in the current study was severe COVID-19 infection leading to ARDS, cardiac arrest and multi-organ failure.

### Prediction of risk factors for severe-critical COVID-19 in patients with and without hypertension

Generally, 23.8% of hypertensive patients developed  severe/critical COVID-19. In order to determine independent predictive variables of severe COVID-19 among hypertensive patients, all variables with statistical significance in univariate distribution were included in logistic regression multivariate analysis. This innovative approach showed that body mass index (BMI) > 25 (OR_Adj_:1.8, 95% CI:1.2 to 2.42; *P*-value: 0.027), age over 60(OR_Adj_:1.26, 95% CI:1.08 to 1.42; P-value: 0.021), increased hospitalization period (OR_Adj_:2.1, 95% CI:1.24 to 2.97; P-value: 0.013), type 2 diabetes (OR_Adj_:2.22, 95% CI:1.15 to 3.31; P-value: 0.001) and chronic kidney disease (OR_Adj_:1.83, 95% CI:1.19 to 2.21; P-value: 0.013) were the significant independent risk factors of severe COVID-19 in hypertensive patients (Table 3). Notably, after performing the same model on the cases without hypertension, it was revealed that only older age was associated with a higher risk of sever disease among these group participants. Severe/critical COVID-19 infection was significantly higher among hypertensive group. Besides, we showed that None of the other risk factors mentioned above (BMI > 25 Kg/m2, CVD, diabetes and chronic kidney disease) are statistically significant among normotensive group. As a result, we can mention hypertension as a risk factor to develop severe COVID-19 infection.

**Table 3:** Multivariate logistic Regression of Factors Associated with Progression of disease in patients with and without hypertension

Variable	group					
	Non-hypertensive			Non-hypertensive		
	OR Adj	95% CI	P value	OR Adj	95% CI	P value
Age>60year-old	1.26	1.08-1.46	0.021	1.06	011. -2.08	0.044
BMI>25(kg/m2)	1.8	1.02–2.42	0.027	1.34	0.88–1.89	0.21
Increased hospital stays (positive)	2.1	1.24–2.97	0.013	1.48	0.36–2.61	0.28
CVD (positive)	3.3	1.3–5.4	0.001	2.8	0.84–4.78	0.058
Diabetes (positive)	2.22	1.15–3.31	0.001	1.24	0.59–1.92	0.28
Kidney daisies (positive)	1.83	1.19–2.21	0.013	1.22	0.66–1.81	0.052

OR _Adj:_ Odds Ratio Adjusted; 95%CI: 95% Confidence Interval

## Discussion

In the present study performed on 598 patients with confirmed COVID-19, 176 (29.4%) patients had underlying hypertension. Using a novel approach, we have showed some important risk factors predictive for developing severe infection among COVID-19 hypertensive patients including Age > 60-year-old, BMI > 25 Kg/m^2^, CVD, diabetes and chronic kidney disease.

As it is proven before, hypertension is the most common underlying disorder among COVID-19 patients [25–29]. Shi et al., and Guo et al., reported a high prevalence of hypertension (59.8–63.5%), among COVID-19 patients which was accompanied by mortality [30, 31]. A study conducted by Lippi et at., reported that hypertension carries higher risk of developing severe COVID-19 [32].

Since hypertension is the most common comorbidity among COVID-19 patients and it has been frequently reported that hypertension increases severity of COVID-19 infection [25, 27–29, 33], a lot of hypertensive people would be worried about ending up with severe COVID-19 infection and poor clinical outcomes, So we have done a completely novel study which can show hypertensive patients the most important risk factors to develop severe COVID-19 among them and tell them should they really be worried about developing severe COVID-19 or not.

There are some studies showing that some laboratory parameters are predictors of worse clinical outcomes in COVID-19 patients. Some of these parameters are cardiac biomarkers, pro-inflammatory cytokines and ferritin [34]. Moreover, some other clinical predictors have also been reported in early studies such as, old age, male gender, cardiovascular diseases, respiratory diseases and diabetes to be related with severe COVID-19 [14] but until this moment, there is no data available to specifically show risk factors of developing severe COVID-19 among hypertensive patients.

The reason why hypertensive patients are at a greater risk of severe COVID-19 is unclear. However, a possible theory is that Human pathogenic coronaviruses (severe acute respiratory syndrome coronavirus family; SARS-CoV, MERS-CoV and SARS-CoV-2), bind to their target cells through Angiotensin Converting Enzyme 2 (ACE2), which is expressed by epithelial cells of the lung, intestine, kidney, and blood vessels [23]. Notably, the underlying hypertension in COVID-19 patients is often treated with angiotensin-converting enzyme inhibitors (ACEI) and angiotensin receptor blockers (ARBs). Controlling hypertension with ACE inhibitors and ARBs, results in an up regulation of ACE2 [35]. Consequently, the increased expression of ACE2 might facilitate COVID-19 infection.

Our novel study reveals predictive factors for developing severe COVID-19 that can be used by physicians to identify high risk hypertensive COVID-19 cases and determine appropriate treatment approach to achieve best possible clinical outcomes.

The only limitation of our study was its retrospective design in which some terms were out of researcher’s control. Further studies in bigger populations and other races are needed to confirm whether our novel predictive factors for developing severe COVID-19 in hypertensive patients can be true about other populations and races or not. Also, all hypertensive patients of this study were receiving anti-hypertensive medications but the data of the exact class of anti-hypertensive medication was not collected.

## Conclusion

Although hypertensive patients are at increased risk of developing more severe COVID-19 compared to normotensives, here we came up with some predictive factors to identify patients with poor outcomes in those with COVID-19 infection which are: Age > 60-year-old, BMI > 25 Kg/m^2^, CVD, diabetes and chronic kidney disorders.

## Data Availability

On presumable requests, the data sets used for analysis during the current study are available from the corresponding author.

## Abbreviations

COVD-19: Coronavirus disease 2019
SARS-CoV-2: Severe acute respiratory syndrome corona virus-2
ACE2: Angiotensin Converting Enzyme 2
ACEI: Angiotensin-converting enzyme inhibitors
ARBs: Angiotensin receptor blockers
OR _Adj_: Odds Ratio Adjusted
